# Comparative transcriptome analysis of inbred lines and contrasting hybrids reveals overdominance mediate early biomass vigor in hybrid cotton

**DOI:** 10.1186/s12864-020-6561-9

**Published:** 2020-02-10

**Authors:** Kashif Shahzad, Xuexian Zhang, Liping Guo, Tingxiang Qi, Huini Tang, Meng Zhang, Bingbing Zhang, Hailin Wang, Xiuqin Qiao, Juanjuan Feng, Jianyong Wu, Chaozhu Xing

**Affiliations:** State Key Laboratory of Cotton Biology, Institute of Cotton Research of Chinese Academy of Agricultural Sciences, Key Laboratory for Cotton Genetic Improvement, Ministry of Agriculture and Rural Affairs, 38 Huanghe Dadao, Anyang, 455000 China

**Keywords:** Heterosis, Hybrid cotton, Biomass vigor, Transcriptome, DEGs, Overdominant, Circadian rhythm

## Abstract

**Background:**

Heterosis breeding is the most useful method for yield increase around the globe. Heterosis is an intriguing process to develop superior offspring to either parent in the desired character. The biomass vigor produced during seedling emergence stage has a direct influence on yield heterosis in plants. Unfortunately, the genetic basis of early biomass vigor in cotton is poorly understood.

**Results:**

Three stable performing F_1_ hybrids varying in yield heterosis named as high, medium and low hybrids with their inbred parents were used in this study. Phenotypically, these hybrids established noticeable biomass heterosis during the early stage of seedling growth in the field. Transcriptome analysis of root and leaf revealed that hybrids showed many differentially expressed genes (DEGs) relative to their parents, while the comparison of inbred parents showed limited number of DEGs indicating similarity in their genetic constitution. Further analysis indicated expression patterns of most DEGs were overdominant in both tissues of hybrids. According to GO results, functions of overdominance genes in leaf were enriched for chloroplast, membrane, and protein binding, whereas functions of overdominance genes in root were enriched for plasma membrane, extracellular region, and responses to stress. We found several genes of circadian rhythm pathway related to LATE ELONGATED HYPOCOTYL (LHY) showed downregulated overdominant expressions in both tissues of hybrids. In addition to circadian rhythm, several leaf genes related to Aux/IAA regulation, and many root genes involved in peroxidase activity also showed overdominant expressions in hybrids. Twelve genes involved in circadian rhythm plant were selected to perform qRT-PCR analysis to confirm the accuracy of RNA-seq results.

**Conclusions:**

Through genome-wide comparative transcriptome analysis, we strongly predict that overdominance at gene expression level plays a pivotal role in early biomass vigor of hybrids. The combinational contribution of circadian rhythm and other metabolic process may control vigorous growth in hybrids. Our result provides an important foundation for dissecting molecular mechanisms of biomass vigor in hybrid cotton.

## Background

Cotton is the prime fiber crop, comprises of more than 50 species, and evolved around 10–20 million years ago (MYA) [1]. The upland cotton (*Gossypium hirsutum* L.) has been cultivated in tropical and temperate regions of the world, contributing 95% of natural textile fiber, and also a substantial source of edible oil. The global population is increasing at an alarming rate, putting agriculture sector under extreme pressure to ensure food security. Another major constraint for sustainable crop production is hasty climate change. To mitigate these challenges, plant breeders are working hard to increase yield and resistant against the pest, disease, biotic and abiotic stresses in crops. In this regard, a major breakthrough was achieved in rice, maize, sunflower, vegetables, and fruits through heterosis breeding. Hybrid rice produced through the utilization of heterosis gave 10–20% more yield than conventional varieties in China [2]. Hybrids of cotton are developed and cultivated mainly in China as well as in India. The development of hybrid cotton at commercial level was not started until the 1980s in China. Since then, hybrids have been harbored with Bt toxin gene as a result area of cultivation was increased after the mid-2000s with the advantage of more yield and resistance [3]. Manual crossing (emasculation and pollination) is one of the major hurdles causing the sluggish pace of hybrid cotton seed development. However, the cytoplasmic male sterile system has been proven an economic tool for commercial hybrid seed production in upland cotton in recent times [4, 5].

Heterosis breeding is not a modern day tool to increase crop yield. In the last century, people had learned crossed fertilized plant produced more output than the self-fertilized. However, the term heterosis was coined by Gorge. H Shull [6]. Heterosis/hybrid vigor is a biological phenomenon to produce superior offspring’s in characteristics of growth, biomass, stature, fertility, disease resistance, and stress management than either parent [7–9]. In contrast, inbreeding depression reveals that continuous self-fertilization declined the crop yield [10]. Allopolyploid cotton contains more than two sets of chromosomes. In spite of this, many researchers reported cotton has significant heterosis in yield contributing and fiber quality traits [11–14]. Interspecific crosses between upland and Pima cotton produce better fiber traits but disease incidence was a major problem. In contrast, intraspecific hybrids gave stability in production, improved fiber traits, and ensure seed purity [15]. In most cases, positive heterosis is required but for plant height, maturity, and gossypol content negative heterosis is desired in cotton.

The genetic basis of crop heterosis have been intensively researched almost for a century with different approaches. Many researchers tried to explain with the so-called hypothesis of dominance [16–18], overdominance [19–21], epistasis [22, 23], and genetic distance [24, 25]. Later on, many studies in agronomic crops provide strong evidence that the genetic basis of heterosis is perturbed. It varies with different species, stages, and traits [26–28]. Generally, crop hybrids show heterosis at two key stages of development e.g. vegetative and reproductive. The biomass produced in the vegetative stage controls the fate of final output, as it provides the energy basis for architecture development and adaptation to adverse conditions. Moreover, early growth advantages increased nutrients acquisition and competitiveness in plants. Leaf area, nodes, and vegetative branches are established during early stage of growth in cotton, and these eventually provide energy basis for the development of fruit branches, flower buds, and bolls. A previous study in cotton reported that during reproductive stage any disturbance in net assimilates can disturb the source and sink direction and finally affect the fiber quality traits [29]. To be concise, any pitfall in vegetative growth directly affects the final output in agronomic crops. Many studies were performed to investigate the molecular basis of vegetative or biomass heterosis in hybrids of rice [30, 31], maize [32, 33], wheat [34], and Brassica [35]. But, less is conducted in cotton. Now, the whole genome sequence of upland cotton is available online to the researchers [36, 37]. Furthermore, high-throughput sequencing technologies have enabled researchers to investigate the molecular mechanism of crop heterosis as a routine task [38, 39].

We designed this study to understand the genetic basis of early biomass vigor in intraspecific hybrid cotton. Most of past studies focused on a single hybrid and both parents to analyze gene expression differences. However, we sequenced root and leaf tissues of three contrasting hybrids together with their inbred parents for better understanding the genetic aspects. Through genome-wide comparative transcriptome analysis, we aimed to identify DEGs, gene expression patterns, and overview the biological pathways that mediate early biomass vigor in cotton. Our result provides a foundation to understand the preliminary biological basis of biomass heterosis. Furthermore, these data resources will be important to find candidate genes of biomass vigor in hybrid cotton in the future.

## Results

### Biomass heterosis exist in contrasting hybrids at the seedling stage

**Fig. 1:**
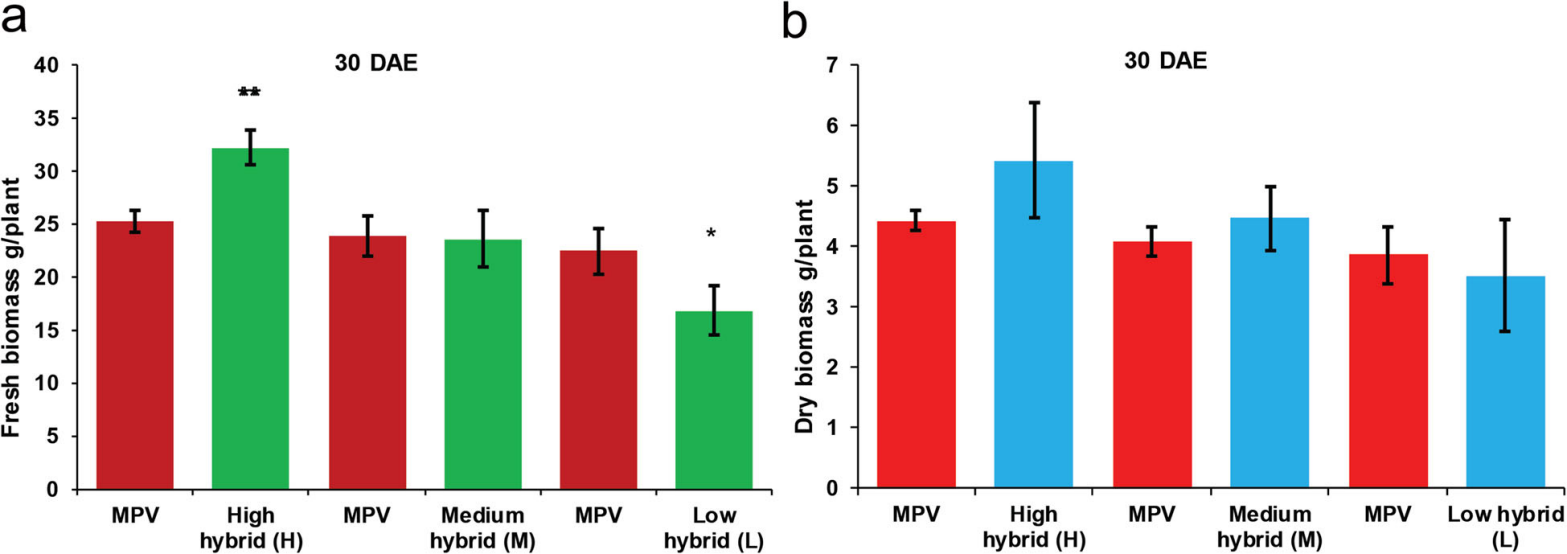
Phenotypic heterosis observed in F_1_ hybrids at 30 days after emergence of seedling (DAE). **a** Fresh biomass observed in hybrids relative to their mid-parent value (MPV). Here ** is used for significant difference at *p* < 0.01 and * at *p* < 0.05. **b** Dry biomass observed in hybrids compared with their MPV

Three contrasting hybrids having high (H), medium (M), and low (L) parent heterosis in yield traits together with their four inbred parents (denoted as A, B, C, and D) were used to investigate the early biomass vigor in the field. We observed high and medium hybrids did not show statistically significant difference in fresh biomass (g/plant) and dry biomass (g/plant) relative to their mid-parent values (MPV) at 10 days after emergence of seedling (DAE) and 20 DAE (Additional file 25: Figure S1 and Additional file 26: Figure S2). However, high hybrid showed highly significant difference in fresh biomass compared with its MPV at 30 DAE (Fig. 1). According to the results, low hybrid showed significant difference in fresh biomass compared with its MPV at 10 DAE (Additional file 25: Figure S1). Similar results were obtained at 20 DAE (Additional file 26: Figure S2) and 30 DAE (Fig. 1). High hybrid showed more biomass accumulation and low hybrid showed decreased biomass accumulation compared to their parents. However, medium hybrid did not show any difference in biomass accumulation compared with both parents. These results indicate biomass heterosis is established in hybrids as compared to their parents during early seedling growth.

### Transcriptome sequencing, mapping and global expressions in root and leaf

Three contrasting F_1_ hybrids and their four inbred parents were used to perform RNA sequencing in this study. Root and leaf tissues of the same plant with three biological replicates were used to build 42 cDNA libraries for RNA Illumina sequencing. The brief detail of sequencing, mapping and gene expressions of the individual library is presented in Additional file 1. The overall sequencing means of valid reads was 98.8% with a value of 90% exon region mapping. The value of Q20% was 99.6% in our sequencing results. In the root of A, B, C, D, H, M, and L genotypes, mean of valid reads was approximately 51, 54, 50, 52, 43, 46, and 45 million, respectively (Table 1). The mean of valid reads in the leaf of A, B, C, D, H, M, and L genotypes was ~ 50, 49, 42, 44, 45, 43, and 45 million, respectively. On an average, more than 89% valid reads were mapped to *G. hirsutum* TM-1 reference genome in this sequencing. However, the mapped percentage in root samples was lower than leaf. The mean of multi-mapped and splice reads was more than 28 and 31%, respectively. In this study, the root tissue of each genotype showed higher number of total expressed genes compared to leaf. For example, approximately 77 thousand genes were expressed in root of A, while it was ~ 75 thousand in leaf (Additional file 1). We also found total numbers of expressed genes in both tissues of F_1_ hybrids were higher than the inbred parents (Additional file 1).

**Table 1:** Comparison results of leaf and root sequences on the reference genome

Samples	Valid reads	Mapped reads	Multi Mapped reads	Non-splice reads	Splice reads
AR	51128076	42941674 (84.1%)	13374298 (26%)	23022576 (45.1%)	15385894 (30.1%)
AL	50223934	48695890 (96.9%)	16157539 (32.1%)	25789688 (51.3%)	17081456 (34%)
BR	54107099	46993196 (86.8%)	14846539 (27.4%)	25014416 (46.2%)	16974977 (31.3%)
BL	49707365	48244852 (97.1%)	16086388 (32.3%)	25040718 (50.3%)	17076845 (34.3%)
CR	50767484	41842045 (82.4%)	13216507 (32%)	22208395 (43.7%)	15152915 (29.8%)
CL	42235064	40631339 (96.2%)	13500391 (32%)	21646516 (51.2%)	13975171 (33.1%)
DR	52834574	41081382 (77.7%)	12835496 (24.3%)	22221619 (42.1%)	14574574 (27.6%)
DL	44050304	42327643 (96.1%)	14086443 (32%)	22479693 (51%)	14580814 (33.1%)
HR	43141279	38301647 (88.8%)	12126751 (28.1%)	20191122 (46.8%)	13615361 (31.1%)
HL	45690950	44124014 (96.5%)	14800421 (32.4%)	23403306 (51.2%)	14810568 (32.3%)
MR	46272200	39103323 (84.8%)	12391214 (26.9%)	20472287 (44.4%)	14045575 (30.5%)
ML	43028428	39441113 (91.8%)	14204489 (32.9%)	20481512 (47.7%)	13220392 (30.8%)
LR	45335083	38959747 (85.9%)	12539366 (27.6%)	20368492 (44.9%)	13919944 (30.7%)
LL	45513687	42998982 (94.7%)	14537458 (31.9%)	22837449 (50.2%)	14431801 (31.7%)

In the table, *R* Root, *L* leaf, *H* high, *M* Medium and *L* Low. A, B, C, and D represents four inbred parents. Mapped reads represent the number of sequences that were mapped to the reference genome. Multi-mapped reads aligned multiple positions on the referenced sequence. Non-splice reads were those that not showed splicing. Spliced reads represent alternative splicing events

### Differentially expressed genes in root and leaf transcriptome

**Fig. 2:**
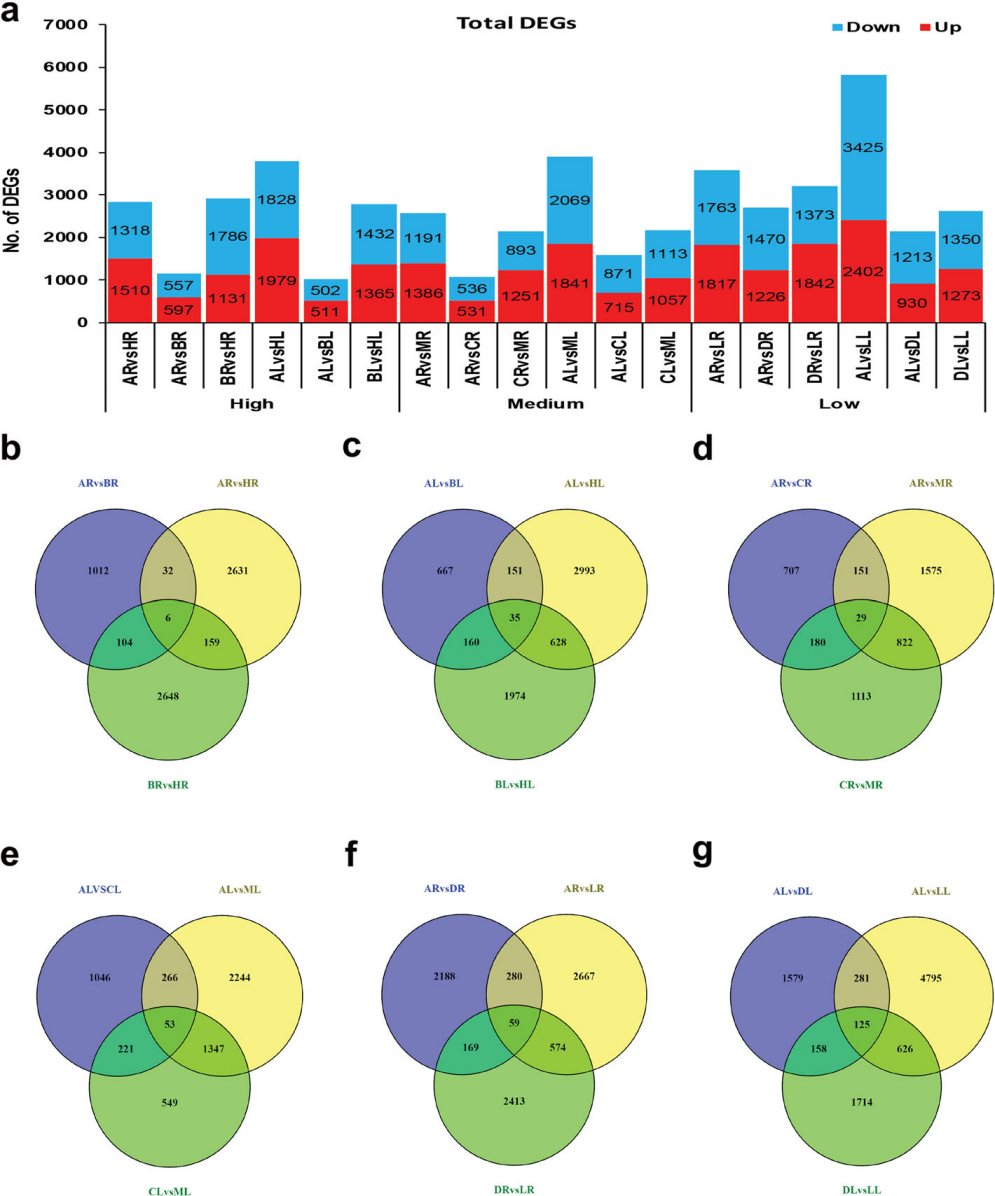
Total DEGs and their distribution in root and leaf of each hybrid parent triad. **a** shows total number of up and down regulated DEGs. Here, R: root, L: leaf, A: maternal parent, and B, C, D represents three paternal parents of high (H), medium (M), and low (L) hybrids, respectively. **b** in root **c** in leaf represents distribution of unique and common DEGs in high hybrid parent triad. Similarly, **d** in root **e** in leaf shows distribution of unique and common DEGs in medium hybrid parent triad. **f** in root **g** in leaf represents unique and common DEGs distribution in low hybrid parent triad

Here, we analyzed the dynamic changes of root and leaf transcriptome in all F_1_ hybrids and their inbred parents. The expression levels significantly different at *p* ≤ 0.05 and with log2 (fold change) > 1 or log2 (fold change) < − 1 designated as differentially expressed genes in each hybrid parent triad. The total number of DEGs (Up + down) among each hybrid parent triad is represented in Fig. 2a. The comparative analysis in the root of hybrid (H) compared with both parents revealed H with maternal parent (A) and paternal parent (B) respectively showed 2917 and 2828 total number of DEGs (Fig. 2a: Additional file 2). The comparison of both parents ARvsBR showed 1154 total number of DEGs. In the leaf transcriptome of H, the comparison of ALvsHL showed 3807 total number of DEGs, whereas the comparison of BLvsHL showed a total of 2797 DEGs (Fig. 2a: Additional file 3). Furthermore, the comparison of both parents ALvsBL showed 1013 total number of DEGs. Distribution of DEGs in H-hybrid revealed major portion of genes was unique and less was common in each tissue (Fig. 2b and c). For instance, 2631 genes in roots and 2993 in leaf were unique in the combination of H with A. Unique DEGs in comparison of B and H was 2648 in root and 1974 in leaf. A large portion of differential gene expression is probably major determinant of better performance in high hybrid.

The root transcriptome of medium hybrid (M) revealed comparison of M with maternal parent (A) and paternal parent (C), respectively showed 2577 and 2144 total number of DEGs (Fig. 2a: Additional file 4). It was observed the comparison of ALvsML had 3910 total number of DEGs in the leaf of M hybrid, whereas the comparison of CLvsML showed 2170 total number of DEGs (Fig. 2a: Additional file 5). Moreover, the combination of both parents had 1067 DEGs in root and 1586 in leaf. Although results of unique and common DEGs distribution in M hybrid identified more unique but less common genes. However, unique DEGs were more in the comparison of M with A than with C (Fig. 2d and e). The comparative analysis revealed total number of DEGs between low hybrid (L) and its maternal parent (A) was 3580 in the root (Fig. 2a: Additional file 6). The combination of L and its paternal parent (D) had 3215 total number of DEGs. Furthermore, the comparison of ALvsLL and DLvsLL, respectively showed 5827 and 2623 total number of DEGs (Fig. 2a: Additional file 7). Distribution of DEGs exposed that the majority of genes were unique, whereas less was overlap (Fig. 2f and g). For example, A versus L had 2647 unique DEGs in root and 4795 in leaf. Unique DEGs in DvsL were 2413 in root and 1714 in leaf. More interestingly, both parents of low hybrid had higher genetic differences among each other than those of medium and high hybrids. Overall, the result of comparative analysis of DEGs between hybrids and parents revealed that hybrids had different genomic constituent relative to their parents. However, comparison of both parents indicated that they have few genetic differences among them.

### F_1_ hybrids exhibited overdominant gene expressions

**Fig. 3:**
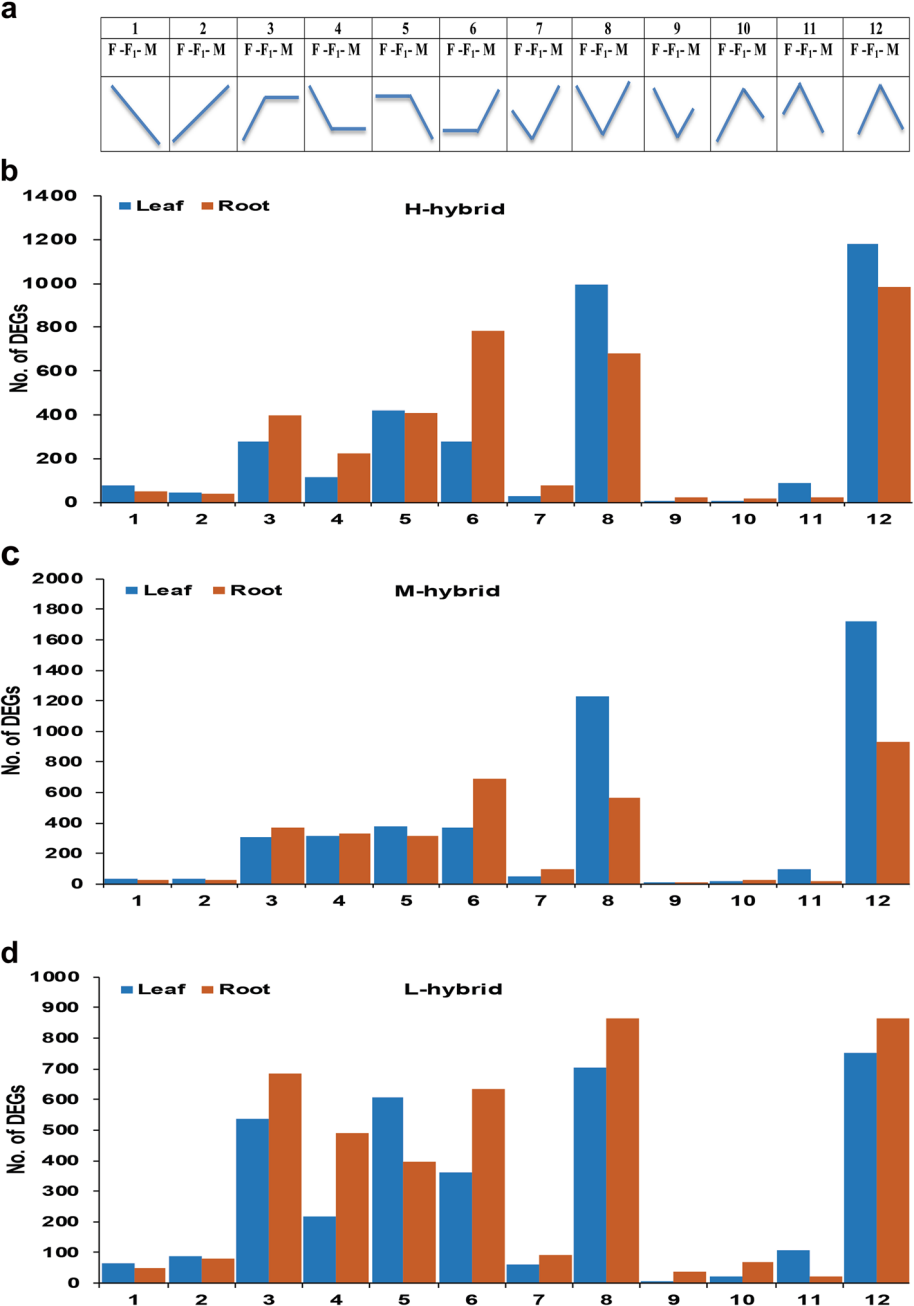
The 12 possible gene expression patterns in F_1_ hybrids compared to their parents in root and leaf. **a** Expression patterns of 12 groups, M: male parent, F_1_: hybrid, and F: female parent. **b** Total number of genes in each group in root and leaf of high (H) hybrid. **c** Total number of genes in each group in root and leaf of medium (M) hybrid. **d** Total number of genes in each group in root and leaf of low (L) hybrid

Allopolyploids have been found to exhibit expression level dominance [35, 40]. So, to address the magnitude and directionality of expressions in interspecific F_1_ upland cotton hybrids, DEGs of root and leaf transcriptome were divided into 12 possible groups as described by Rapp et al.*,* [41]. Genes in groups 1–2 showed additive expression. The expression of genes in groups 3–4 was termed as male expression level dominance (M-ELD), wherein the expression of genes in groups 5–6 was named as female expression level dominance (F-ELD). The expression of genes in groups 7–9 called downregulated overdominance, wherein the expression of genes in groups 10–12 was named as upregulated overdominance (Fig. 3a). The result of expression patterns analysis in high hybrid (H) in both tissues detected limited number of genes was additive expressions. Male and female parent like expression groups also had few genes. However, it was found overdominant upregulated (group12) and downregulated (group 8) groups had the highest number of genes in both tissues (Fig. 3b: Additional file 8). The analysis results of medium hybrid (M) also dissected overdominant up and downregulated groups had the highest number of genes in both tissues (Fig. 3c: Additional file 9). The analysis result of low hybrid (L) was also similar to H and M hybrids (Fig. 3d: Additional file 10). However, groups of parent-like expression also had many genes in L hybrid as compared to H and M hybrids. The result of expression patterns analysis directed that overdominance at the gene expression level contributes to early biomass vigor in hybrid cotton.

### Functional annotations of overdominant genes

**Fig. 4:**
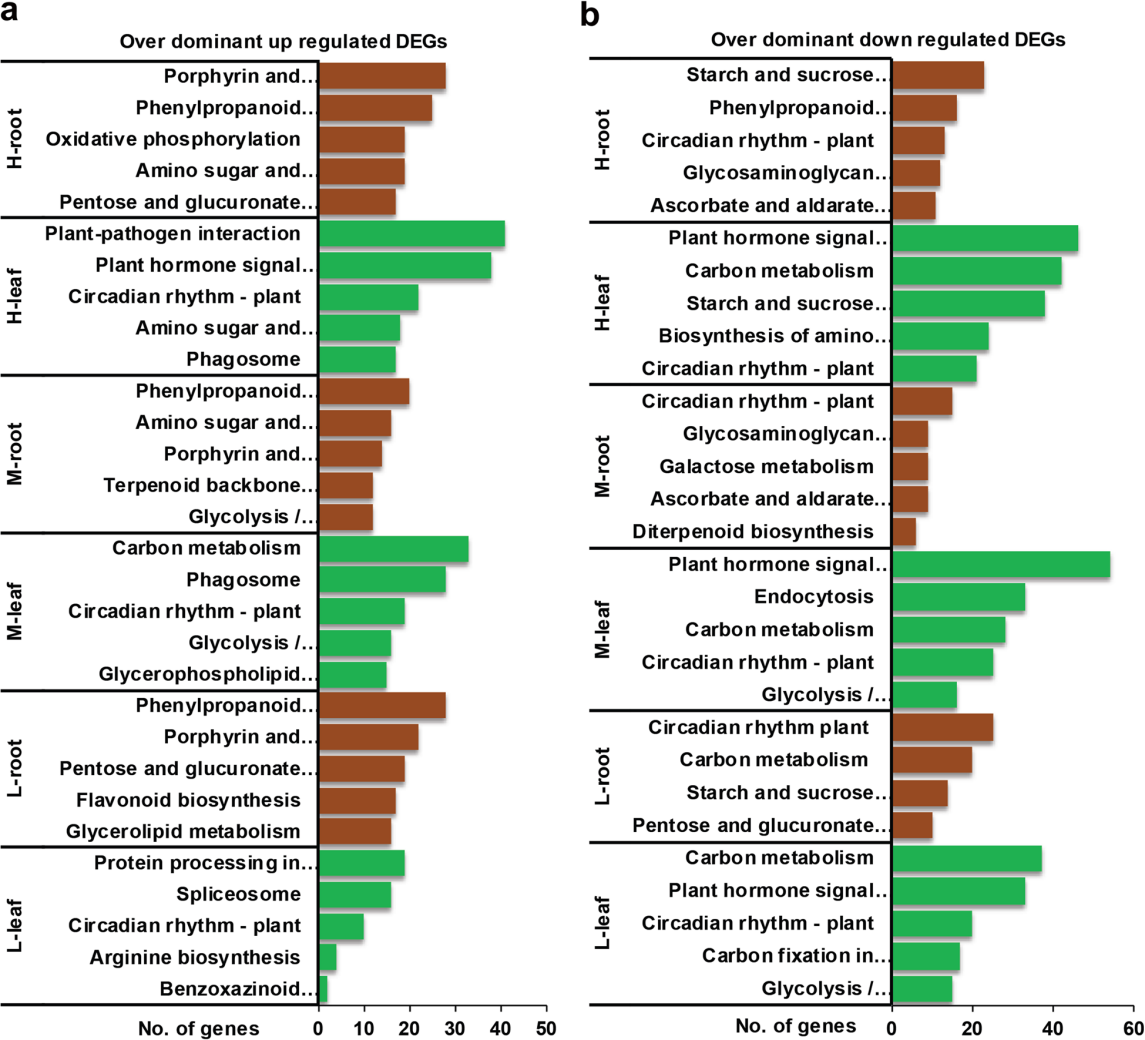
Enriched pathways of overdominant DEGs in root and leaf of F_1_ hybrids. H: High, M: Medium, and L: Low **a** Pathways of upregulated overdominant DEGs. **b** Pathways of downregulated overdominant DEGs

To understand the functions of genes with overdominant expressions in biomass heterosis, GO and KEGG enrichment analysis was implemented in root and leaf of hybrids. GO enrichment analysis (*p-value* < 0.01) of overdominant genes in root of high hybrid (H) relative to its parents revealed most of the upregulated genes were involved in functions related to plasma membrane, regulation of transcription/DNA-template, and extracellular region. Conversely, downregulated genes were enriched in plasmodesma, integral component of plasma membrane, and vacuole (Additional file 27: Figure S3a, Additional file 11). KEGG pathway enrichment analysis (*p-value* < 0.05) of overdominant genes showed most of the upregulated genes were enriched in porphyrin & chlorophyll metabolism, phenylpropanoid biosynthesis, and oxidative phosphorylation pathways (Fig. 4a: Additional file 12). But, the majority of downregulated genes were enriched in starch & sucrose metabolism, phenylpropanoid biosynthesis, and circadian rhythm plant (Fig. 4b: Additional file 12). In the leaf of H hybrid, most of the overdominant upregulated genes were involved in functions of plasma membrane, protein serine/threonine kinase activity, and plasmodesma, while downregulated genes were involved in cellular functions of chloroplast, membrane, and extracellular region (Additional file 27: Figure S3a: Additional file 13). Enriched pathways of overdominant genes found upregulated genes were enriched in plant-pathogen interaction, plant hormone signal transduction, and circadian rhythm plant (Fig. 4a: Additional file 14). In contrast, significant pathways for downregulated genes were plant hormone signal transduction, carbon metabolism, and circadian rhythm plant (Fig. 4b: Additional file 14).

The results of GO enrichment analysis of overdominant genes in the root of medium hybrid (M) compared with its parents uncovered that upregulated genes were enriched in plasma membrane, extracellular region and oxidation-reduction process. In contrast, downregulated genes were involved in following functions e.g. integral component of the plasma membrane and response to salt stress (Additional file 27: Figure S3b: Additional file 15). Pathway enrichment analysis of overdominant genes found upregulated genes were enriched in phenylpropanoid biosynthesis, amino & nucleotide sugar metabolism, and porphyrin & chlorophyll metabolism (Fig. 4a: Additional file 16). For downregulated genes, enriched pathways were circadian rhythm plant, glycosaminoglycan degradation, and ascorbate and aldarate metabolism (Fig. 4b: Additional file 16). GO enrichment analysis of overdominant genes in leaf of M hybrid revealed most of upregulated genes performed functions related to chloroplast, chloroplast stroma, and plastid. However, downregulated genes involved in the following functions such as chloroplast, protein binding, and ATP binding (Additional file 27: Figure S3b: Additional file 17). Pathway enrichment analysis of overdominant genes revealed most of upregulated genes were found in carbon metabolism, phagosome, and circadian rhythm plant (Fig. 4a: Additional file 18). On the other hand, downregulated genes were enriched in planted hormone signal transduction, endocytosis, and carbon metabolism (Fig. 4b: Additional file 18).

GO enrichment analysis of overdominant genes in the root of low hybrid (L) compared with its parents found following enriched terms e.g. plasma membrane, extracellular region, and oxidation-reduction process. The functions of downregulated genes were enriched for chloroplast, regulation of transcription/DNA-template, and transcription factor activity/sequence-specific DNA binding (Additional file 27: Figure S3c: Additional file 19). Enriched pathways of overdominant upregulated genes were phenylpropanoid biosynthesis, porphyrin & chlorophyll metabolism, and pentose & glucuronate interconversions (Fig. 4a: Additional file 20). On the other hand, most of downregulated genes were enriched in circadian rhythm plant, carbon metabolism, and starch & sucrose metabolism (Fig. 4b). In the leaf of L hybrid, GO enrichment analysis of overdominant genes showed functions of upregulated genes were enriched in chloroplast, protein folding, and embryo development ending in seed. In contrast, majority of the downregulated genes were involved in cellular function of chloroplast, membrane, and chloroplast stroma (Additional file 27: Figure S3c: Additional file 21). The pathway enrichment analysis of overdominant genes uncovered majority of upregulated genes were involved in protein processing in the endoplasmic reticulum, spliceosome, and circadian rhythm plant (Fig. 4a: Additional file 22). For downregulated genes, enriched pathways were carbon metabolism, plant hormone signal transduction, and circadian rhythm plant (Fig. 4b: Additional file 22).

### The comparison of overdominance genes between contrasting hybrids

**Fig. 5:**
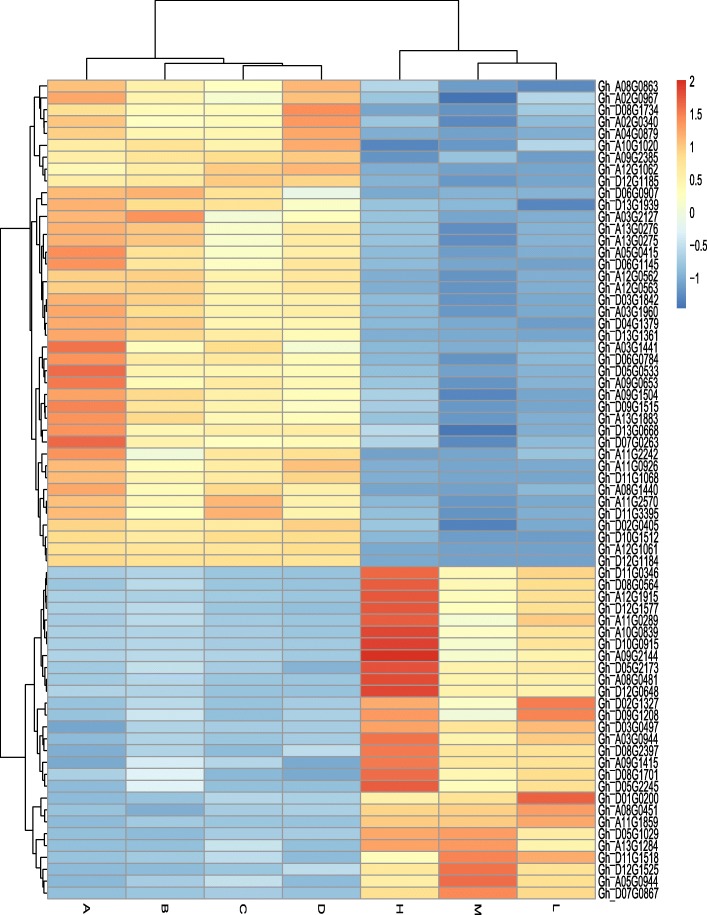
Expression heatmap of all common overdominance genes of F_1_ hybrids. H: High, M: Medium, L: Low, A, B, C, and D represent four inbred parents

The results of KEGG revealed that most of the overdominant genes of high, medium and low hybrids relative to their parents were enriched in similar pathways. We performed comparison analysis between high, medium and low hybrids to see unique and common overdominant expressed genes in the above mentioned pathways. The results showed that 44 genes in leaf and 29 genes in root were common in comparison of all hybrids (Additional file 28: Figure S4: Additional file 23). On the other hand, many genes were also overlapped between comparisons of two hybrids. The reason behind these results might be the common female parent in hybrids. The expression level of genes that showed overdominant expression in all hybrids relative to their inbred parents is presented in Fig. 5. We found most of root genes were belonging to circadian rhythm, phenylpropanoid biosynthesis, and porphyrin & chlorophyll metabolism, while leaf genes were enriched in circadian rhythm, plant hormone signal transduction, and carbon metabolism. Majority of these genes were involved in functions related to DNA binding, oxidation-reduction process, heme binding, and response to oxidative stress (Additional file 29: Figure S5). We presumed phenomenal changes in these pathways associated genes may be transformed biomass vigor in hybrids of cotton.

### The circadian rhythm, metabolic processes, and biomass vigor

Through compression analysis between hybrids, we found circadian rhythm plant pathway contained many overdominant genes in root and leaf of all hybrids. Genes in this pathway had tissue-specific expression except of three (*Gh_A12G1061, Gh_A12G1062,* and *Gh_D12G1185*)*.* In this pathway, six genes (*Gh_A11G0926, Gh_A12G1061, Gh_A12G1062, Gh_D12G1185, Gh_D12G1184,* and *Gh_D11G1068*) related to MYB domain transcription factor, encoding LHY protein showed downregulation in root of hybrids (Additional file 30: Figure S6). In contrast, five genes (*Gh_A12G1061, Gh_A12G1062, Gh_D12G1185, Gh_A09G1504*, and *Gh_D09G1515*) encoding LHY protein showed downregulated expression in leaf of hybrids. The results indicated four genes (*Gh_A08G0451, Gh_D01G0200, Gh_D07G0867,* and *Gh_D11G1518*) associated with transcript factor CO-like showed upregulation in leaf of hybrids (Additional file 30: Figure S6). Genes (*Gh_A05G0944* and *Gh_D05G1029*) named as CIA2 (CHLOROPLAST IMPORT APPARATUS 2) associated with PSEUDO-RESPONSE REGULATOR9 (PRR9) also showed similar results in leaf of hybrids (Additional file 29: Figure S5). In addition to circadian rhythm, several genes (*Gh_A03G0944, Gh_A09G1415, Gh_A11G1859, Gh_A12G1915,* and *Gh_D02G1327*) in phenylpropanoid biosynthesis related to peroxidase superfamily protein, and many genes from porphyrin &chlorophyll metabolism showed upregulated expressions in root of hybrids. Furthermore, many genes of plant hormone signal transduction pathways linked to AUX/IAA transcriptional regulator family protein and genes from carbon metabolism pathway also found with downregulated expressions in leaf of hybrids. To be concise, all these results suggest genes in circadian rhythm together with other metabolic process performed overdominance that might change root and leaf growth, and ultimately lead to altered biomass vigor in hybrid cotton.

### qRT-PCR analysis

**Fig. 6:**
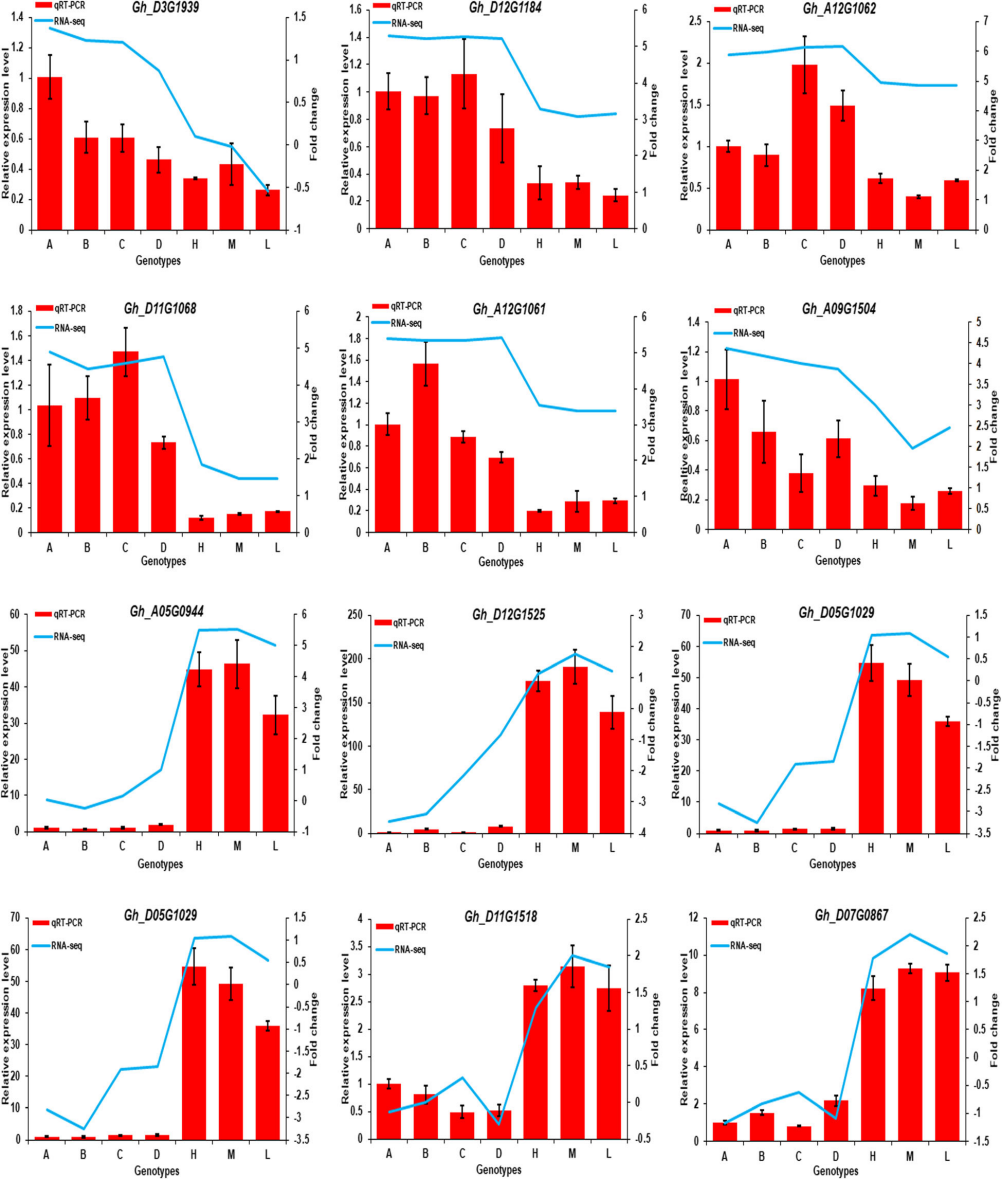
qRT-PCR of 12 DEGs involved in the circadian rhythm pathway and showed overdominance performance in all hybrids relative to their parents. In each figure, H: High, M: Medium, L: Low and A, B, C, and D represents four inbred parents. First five genes represent expression in root, while later genes represent expression in leaf

Twelve overdominant genes from circadian rhythm plant were selected to validate RNA-seq data by real-time qRT- PCR analysis. All selected genes had overdominance expression in high, medium, and low hybrids relative to their parents in RNA-seq results. We selected five genes (*Gh_A12G1061, Gh_A12G1062, Gh_D12G1184, Gh_D11G1068,* and *Gh_D13G1939*) from root and one gene (*Gh_A09G1504*) from leaf with downregulated expressions in hybrids, while six genes (*Gh_A08G0451, Gh_D01G0200, Gh_D11G1518, Gh_D05G1029 Gh_D07G0867, Gh_A05G0944, and Gh_D12G1525*) were selected from leaf with upregulated expressions in hybrids. It was found that the expression trends as calculated by qRT-PCR were consistent with RNA-seq data, thus confirming the accuracy of the RNA-seq results in this study (Fig. 6).

## Discussion

### Overview of early biomass vigor and transcriptome sequence

Heterosis refers to the superiority of offspring in the desired character or traits than either parent. Hybrid breeding effectively utilized the magic of heterosis to produce better quality and quantity of crops worldwide. Both intraspecific and interspecific crosses of cotton showed heterosis in yield contributing traits. However, success of significant heterosis in intraspecific crosses of upland cotton is not an easy task for breeders. Despite different complexities, our team has successfully developed nine upland cotton hybrids. To be honest, researchers are working hard to resolve the complex genetic basis of crop heterosis. Still, a lot of efforts are required particularly in the complex genome of tetraploid cotton. The biomass produced during the early stages of growth plays significant role in yield increments in plants. Keeping this in view, we performed this comprehensive study to investigate the genetic basis of early biomass heterosis in hybrid cotton by comparative transcriptome analysis of contrasting hybrids and their inbred parents.

In this study, three intraspecific upland cotton hybrids varying in yield heterosis named as high (H), medium (M) and low (L) hybrids and their inbred parents were used to investigate early biomass heterosis. According to the results of the field evaluation, these hybrids showed phenotypic heterosis in biomass accumulation than both parents during the early stage of seedlings growth. More biomass accumulation in high hybrid possibly related to more efficiency in nutrient uptake and photosynthetic process. Moreover, early growth advantages seem to be important to achieve heterosis in other traits. A previous research found intraspecific hybrid plants produced extreme phenotypes than both parents in the diversity of traits such as morphology, physiology, and biochemical attributes of tissues or organs [42]. A study in rice measuring the seedling traits observed that hybrids showed mid and better parent heterosis in all seedling traits expect height [30]. Wheat hybrids showed strong hybrid vigor compared to both inbred lines during the seedling stage [34]. Increased cotyledon size was recorded even a few days after sowing in Arabidopsis hybrids [43].

As a genetic perspective, the root and leaf transcriptome sequencing was performed to generate large scale cDNA sequence data and profile transcriptome changes in contrasting hybrids and their inbred parents. The trancriptomic data uncovered that total number of expressed genes were somehow higher in hybrids than all parents especially in root. More expressed genes in hybrids may have a relationship with an altered role in the diversity of key functions including anchorage the plant, absorption of water and nutrients, photosynthetic process, and many other metabolic processes. Root arises first from radical, so more expressed genes may be having a correlation with more functional changes during the early stage. The root vigor during early stages of seedling growth has been documented in hybrids of maize and wheat [44, 45]. In this study, comparative analysis among hybrids and parents showed many DEGs in hybrids compared with either parent in both root and leaf tissues. However, comparisons of parents had only limited number of DEGs, thus indicating similarity in their genomic constituent. These results predict crossing of genetically similar inbred lines leads to changes in the genetic organization of hybrids. At the early stages of growth, these changes caused differential expression of genes as a consequence biomass heterosis has witnessed in hybrids. The results of many recent studies reported differences in genomic expressions between hybrids and parental lines have a direct relationship with the level of heterosis [46, 47]. Therefore, superior performance is supposed to be the result of global differences of gene expressions between homozygous inbred lines and heterozygous hybrids.

### Overdominant gene expressions mediate early biomass vigor in cotton

To assess the gene expression patterns in hybrids compared with parents, the expressions of DEGs in F_1_ hybrids are statistically divided into 12 groups. We observed expression of few genes was additive in both tissues of hybrids. The male or female parent like expression groups also had few numbers of genes. In contrast to our results, it was stated in cotton that synthetic and natural allopolyploids showed expression level dominance and follow the expression patterns of one of two diploids parents [41, 48]. However, Yoo et al.*,* [40] suggested that these changes became more balanced in natural allopolyploid cotton on evolutionary timescale by higher rates of novel gene expression patterns as well as homoeolog silencing. They also found domesticated cotton had more novel expressed genes than its wild mate. Therefore, provides striking evidence that strong artificial selection leads to new gene expression patterns in crops. In our study, the expression patterns of most DEGs were overdominant in both tissues of contrasting hybrids. These results demonstrated that there are higher expressions of heterozygous alleles in the hybrids that altered the biomass vigor in hybrids relative to their parents. Previously, Zhao et al.*,* [49] researched the mechanism of cotton heterosis during four different growing and developmental stages. They observed quantitative and qualitative gene expressions between hybrids and parents and suggested overdominant gene expressions had a positive influence on heterosis. Incomplete dominance of deleterious alleles contributes extensively to trait variation and heterosis in maize [50]. In rice, a study to dissect the genetic basis of 38 agronomic traits concluded numerous superior alleles with positive dominance effect caused heterotic phenotypes [51]. However, a recent study reported allelic specific expression or imbalance expression of two parental alleles in hybrids caused heterosis in rice [52].

### Circadian clock and metabolic genes play critical role in early biomass vigor of cotton

To a particular interest, the functions of most of the overdominant genes in hybrids were enriched to chloroplast, protein binding, plasma membrane, extracellular region, and responses to stress. The result of KEGG analysis exposed most of the overdominant genes of contrasting hybrids relative to their parents were enriched in similar pathways. These pathways were circadian rhythm, phenylpropanoid biosynthesis, porphyrin & chlorophyll metabolism, plant hormone signal transduction, and carbon metabolism. Interestingly, many genes involved in circadian rhythm plant had overdominant expressions in hybrids in both root and leaf. qRT-PCR results also verify this (Fig. 6). Circadian clock controls biological processes of plants through transcriptional and post transcriptional regulation of many output processes [53, 54]. For example, genes expression changes in circadian rhythm have an influence on photosynthesis, carbohydrate, and accumulation of chlorophyll, starch and sugar. Through repression facilitated by evening loop protein complex, this clock also regulates hypocotyl growth and flowering in plants [55]. Many genes in our study related to MYB domain transcription factor, LHY encoding protein showed downregulated expressions in both tissues of hybrids. In contrast, two genes named CIA2 had upregulated expressions in leaf. These two genes are associated with morning loop protein complex known as PRR9. These results predict that changes in circadian clock-associated genes might lead to the more rapid growth of root and leaf in hybrids compared with parents.

It is a well-known fact that in the morning-phased loop, LHY and CCA1 (CIRCADIAN CLOCK ASSOCIATED 1) activate the expression of PSEUDO-RESPONSE REGULATOR 7 (PRR7) and PRR9 genes to maintain clock length and amplitude in plants [56]. Ni et al.*,* [57] reported that during the day, epigenetic repression of CCA1 and LHY induced the expression of TIMING OF CAB EXPRESSION1 (TOC1), GIGANTEA (GI), and downstream genes that contain evening elements. These caused changes in circadian-mediated physiological and metabolic pathways. As a result, growth and biomass vigor were produced in allotetraploids and F_1_ hybrids of Arabidopsis. Overexpression of ZmCCA1b in maize disturbed circadian rhythms resulting in decreased chlorophyll content, plant height, and node elongation of stems thus, biomass heterosis was altered [58]. In our study, four genes associated with transcript factor CO-like showed upregulated expressions in the leaf of hybrids. These genes are believed to be involved in flowering time in response to daylight. Light responsive circadian regulators switch vegetative growth to flowering and maybe this resulting in different level of biomass vigor in hybrids. Such type of regulator in rice named as *Ghd7* affects plant height, heading date, and final yield [59]. Furthermore, CO-like genes control flowering in response to day length in Arabidopsis [60, 61]. According to our results, clock associated genes showed differential expression in a tissue-specific way as these were not common between root and leaf. Previous experimentation in Arabidopsis reported roots and shoots circadian clock is clearly different from each other, therefore plant clock is organ specific [62].

In addition to circadian rhythm, other pathways, such as plant hormone signal transduction, carbon metabolism, phenylpropanoid biosynthesis, and porphyrin & chlorophyll metabolism may also contribute to biomass vigor in cotton. As, altered expression of circadian rhythm genes also disturbs the other biological pathways such as phytohormone signaling, stress response, and energy metabolism [63]. We found many genes of AUX/IAA transcriptional regulator family protein with downregulated expressions in leaf of hybrids. Auxin hormone plays a diversity of functions in plants. It regulates leaf initiation, leaf shape, and shoots apical meristems. Genes involved in IAA biosynthesis showed decreased expression in *B. napus* hybrids and play role in hybrid vigor [27]. According to a study in Arabidopsis, the gene activity of IAA was frequently increased in hybrids that promote leaf cell numbers [64]. Furthermore, many genes of peroxidase superfamily protein showed upregulated expression in root of hybrids in our results. We assume changes in these genes may be contributing to root growth in hybrids. Peroxidases are thought to be involved in a variety of physiological functions in plants e.g. lignification, suberization, cross-linking of cell wall proteins, and wound healing. A previous study has shown peroxidase participates in auxin metabolism [65]. Cell wall related peroxidase effect the root elongation in Arabidopsis [66]. Taken together all, our result predict genes involved in circadian rhythm and other metabolic processes showed overdominance in hybrids resulting in altered biomass vigor in cotton.

## Conclusions

It is notable that F_1_ hybrids of upland cotton showed biomass vigor during the early stages of vegetative growth. The genome-wide comparative transcriptome study of hybrids and inbred lines determined many DEGs compared to either parent in root and leaf of hybrids. Further analysis indicated expression pattern of most DEGs was overdominant in both tissues of hybrids. These results strongly suggest at expression level overdominance mediate early biomass vigor in hybrids of cotton. Phenomenal changes in genes of circadian rhythm and other metabolic processes may be enabled vigorous growth of hybrids relative to their parents. However, how overdominant expressed genes involved in these pathways lead to the different level of biomass heterosis in hybrids needed further investigation. Our comprehensive study gave new insights to understand the preliminary molecular mechanism of biomass heterosis. Our results and large scale data will be potential source for further molecular research on biomass vigor in hybrid cotton.

## Methods

### Plant materials and estimation of early biomass heterosis

In 2015, our research group crossed five maternal and six paternal inbred lines to produce 30 intrapecific upland cotton hybrids with North Carolina mating design II (NCII). After two-year field experimentation at three locations, the level of heterosis for boll number per plant, boll weight, lint percentage, lint yield, and seed cotton yield were investigated in hybrids and parents. Results can be seen in our previous study [67]. According to results of the field test, when one maternal inbred line named SJ48 (denoted as A) crossed with three paternal inbred lines viz. Z98–15 (B), 851–2 (C), and DT-8 (D), these three crosses constantly produced high, medium, and low heterosis in all yield contributing traits, respectively. We selected these three stable performing F_1_ hybrids (H, M, and L) and their inbred parents for biomass study. In 2018, all seven genotypes were planted at the east cotton breeding farm of Cotton Research Institute of the Chinese Academy of Agricultural Science, Anyang, China. Randomized complete block design with three replications was used to arrange plant materials. Seeds were sown in late April. To overview phenotypic biomass heterosis, we harvested five plants and consider each plant as a replicate from each genotype on 10 days after emergence of seedling (DAE), 20 DAE, and 30 DAE. Fresh and dry biomass were calculated for each genotype on these intervals. Fresh biomass of the whole plant was measured immediately after harvesting. The dry biomass of the whole plant was calculated after overnight oven drying at 100 °C.

### RNA extraction, Illumina sequencing, and data analysis

For transcriptomic analysis, total RNA was extracted from each genotype. For RNA extraction, seedlings were raised in standard Hoagland solution in the lab. When true leaves were fully expanded then root and leaf tissue of the same plant was harvested in three biological replications. All harvested samples were instantly placed in liquid nitrogen and stored at − 80 °C before use. Total RNA was isolated using the Sigma Spectrum Plant Total RNA kit (Sigma-Aldrich, USA) according to the manufacturer’s protocol. The total RNA quantity and purity were analyzed on Bioanalyzer 2100 and RNA 6000 Nano LabChip Kit (Agilent, CA, USA) with RIN number > 7.0. Then the cleaved RNA fragments were reverse-transcribed to create the 42 individual final cDNA libraries (21 for root and 21 for leaf tissue for each of the seven genotypes) in accordance with the protocol for the mRNA-Seq sample preparation kit (Illumina, San Diego, USA), the average insert size for the paired-end libraries was 300 bp (±50 bp). The paired-end sequencing on an Illumina Hiseq 4000 was performed at the (LC-bio, China) following the vendor’s recommended protocol. Before assembly, reads that contained adaptor contamination, undetermined bases, and low-quality bases were removed. HISAT2 [68] was used to map reads to the genome of (http://mascotton.njau.edu.cn/info/1054/1118.htm). The mapped reads of each sample were assembled using StringTie [69]. The final transcriptome was generated using Perl scripts. StringTie [69] and Ballgown [70] were used to calculate the expression levels of all transcripts. StringTie [69] was used to perform expression level for mRNAs by calculating FPKM. The differentially expressed mRNAs were selected with log2 (fold change) > 1 or log2 (fold change) < − 1 and with statistical significance (*p*-value < 0.05) by R package Ballgown [70]. The expression level dominance analysis was performed by using EdgeR software as described by Rapp et al.*,* [41]. GO enrichment and KEGG pathway enrichment analysis was performed on the DEGs using the Goatools software (https://github.com/tanghaibao/Goatools) and KOBAS software (http://kobas.cbi.pku.edu.cn) [71], with a corrected *p*-value ≤0.05 as the threshold and rich factor.

### qRT-PCR analysis

For, quantitative real-time RT-PCR, we used the same total RNA to generate first standard cDNA that was used in RNA sequencing. PrimerScript RT Reagent Kit (Perfect Real Time, TaKaRa, Japan) was used to generate cDNA for each replication. Primers for qPCR were designed using the Oligo7 software, synthesized commercially (Biotechnology, Beijing, China), and are shown in Additional file 24. Protocol of qRT-PCR was same as described in our previous study [4]. Three biological replicates were used for each sample and the actin gene was used for normalization. Gene relative expression levels were calculated using the 2^−ΔΔCt^ method.

## Supplementary information

**Additional file 1.** Brief detail of sequencing, mapping and gene expressions of the individual library

**Additional file 2.** Information on DEGs in root of high hybrid-parent triad

**Additional file 3.** Information on DEGs in leaf of high hybrid-parent triad

**Additional file 4.** Information on DEGs in root of medium hybrid-parent triad

**Additional file 5.** Information on DEGs in leaf of medium hybrid-parent triad

**Additional file 6.** Information on DEGs in root of low hybrid-parent triad

**Additional file 7.** Information on DEGs in leaf of low hybrid-parent triad

**Additional file 8.** Information on gene expression patterns in root and leaf of high hybrid relative to its parents

**Additional file 9.** Information on gene expression patterns in root and leaf of medium hybrid relative to its parents

**Additional file 10.** Information on gene expression patterns in root and leaf of low hybrid relative to its parents

**Additional file 11.** Enriched GO terms of overdominant genes in root of high hybrid-parent triad

**Additional file 12.** Enriched KEGG terms of overdominant genes in root of high hybrid-parent triad

**Additional file 13.** Enriched Go terms of overdominant genes in leaf of high hybrid-parent triad

**Additional file 14.** Enriched KEGG terms of overdominant genes in leaf of high hybrid-parent triad

**Additional file 15.** Enriched Go terms of overdominant genes in root of medium hybrid-parent triads

**Additional file 16.** Enriched KEGG terms of overdominant genes in root of medium hybrid-parent triad

**Additional file 17.** Enriched Go terms of overdominant genes in leaf of medium hybrid-parent triad

**Additional file 18.** Enriched KEGG terms of overdominant genes in leaf of medium hybrid-parent triad

**Additional file 19.** Enriched Go terms of overdominant genes in root of low hybrid-parent triad

**Additional file 20.** Enriched KEGG terms of overdominant genes in root of low hybrid-parent triad

**Additional file 21.** Enriched Go terms of overdominant genes in leaf of low hybrid-parent triad

**Additional file 22.** Enriched KEGG terms of overdominant genes in leaf of low hybrid-parent triad

**Additional file 23.** Information of overdominant genes those were unique and common among comparisons of high, medium and low hybrids in root and leaf

**Additional file 24.** List of Primers for quantitative RT-PCR

**Additional file 25: Figure S1.** Phenotypic heterosis observed in all F_1_ hybrids at 10 days after emergence of seedling (DAE). a Fresh biomass in hybrids compared with their mid-parent value (MPV). b Dry biomass observed in hybrids compared with their MPV.

**Additional file 26: Figure S2.** Phenotypic heterosis observed in all F_1_ hybrids at 20 days after emergence of seedling (DAE). Here * is used for significant difference at *p* < 0.0 a Fresh biomass in hybrids compared with their mid-parent value (MPV). b Dry biomass observed in hybrids compared with their MPV.

**Additional file 27: Figure S3.** Most enriched GO terms of overdominant DEGs in root and leaf of F_1_ hybrids. a, b, and c shows GO terms with total number of genes for up and down overdominant DEGs of high (H), medium (M) and low (L) hybrids, respectively. Here, most enriched GO terms with *p* < 0.05 are only presented.

**Additional file 28: Figure S4.** Venn diagram representing the comparison of overdominant genes between hybrids in root and leaf. L: Leaf, R: Root, H, M, and L represent high, medium, and low hybrids respectively. a Distribution of genes in root. b Distribution of genes in leaf.

**Additional file 29: Figure S5.** Enriched GO terms for genes that showed overdominant expressions in all hybrids relative to their parents in root and leaf.

**Additional file 30: Figure S6.** Functional annotations and mode of regulation for overdominant DEGs involved in circadian rhythm plant pathway. This figure is an interpretation of online available figure (https://www.kegg.jp/kegg-bin/show_pathway?ko04712+K12133).

## Data Availability

The datasets generated or analyzed in this study are freely available with accession number GSE144676 at https://www.ncbi.nlm.nih.gov/geo/query/acc.cgi?acc=GSE144676.

## Abbreviations

CCA1: Circadian clock associated1
CO-LIKE: Constans-like
DAE: Days after emergence
DEGs: Differentially expressed genes
F-ELD: Female expression level dominance;
H: High
L: Low
LHY: Late elongated hypocotyl
M: Medium
M-ELD: Male expression level dominance
MPV: Mid parent value
PRR9: Pseudo-response regulator 9
