# Supplementary figures and images for: Comparative transcriptome analysis of inbred lines and contrasting hybrids reveals overdominance mediate early biomass vigor in hybrid cotton
Source: BMC Genomics. 2020 Feb 10;21:140. doi: 10.1186/s12864-020-6561-9 (PMC7011360; doi:10.1186/s12864-020-6561-9)

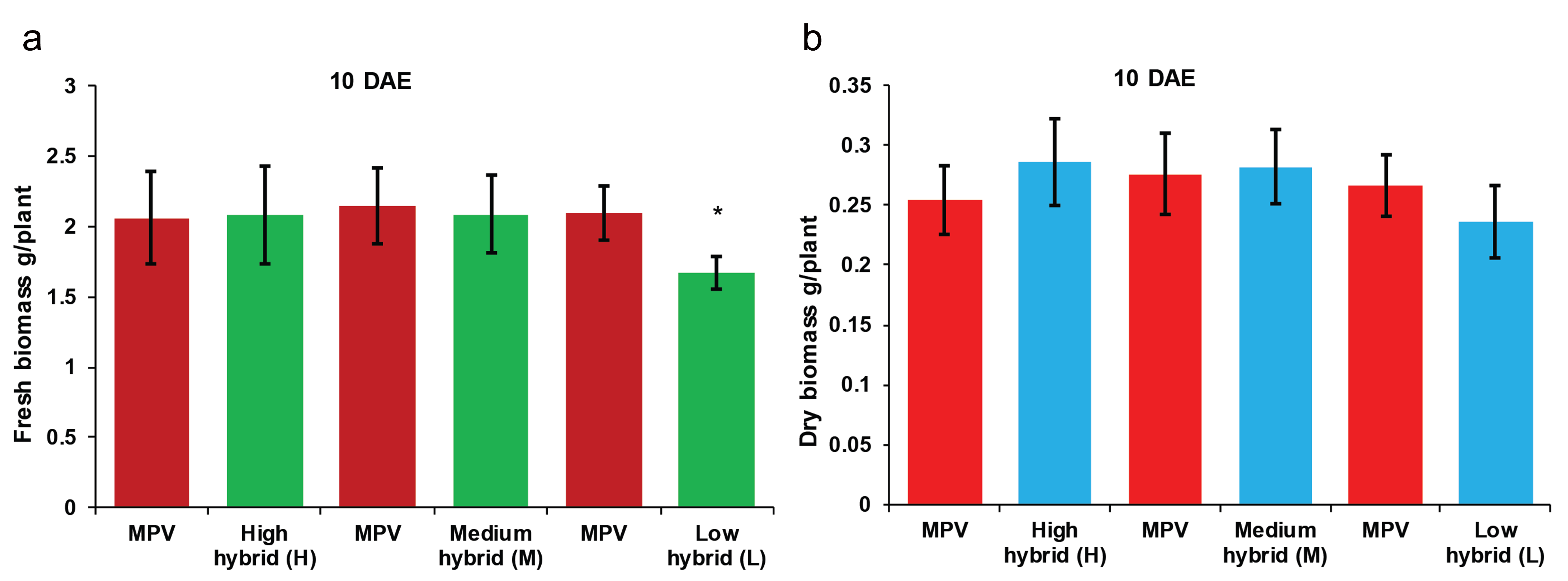

Supplement: Supplementary file 25 — Additional file 25: Figure S1. Phenotypic heterosis observed in all F1 hybrids at 10 days after emergence of seedling (DAE). a Fresh biomass in hybrids compared with their mid-parent value (MPV). b Dry biomass observed in hybrids compared with their MPV. [file 12864_2020_6561_MOESM25_ESM.png]

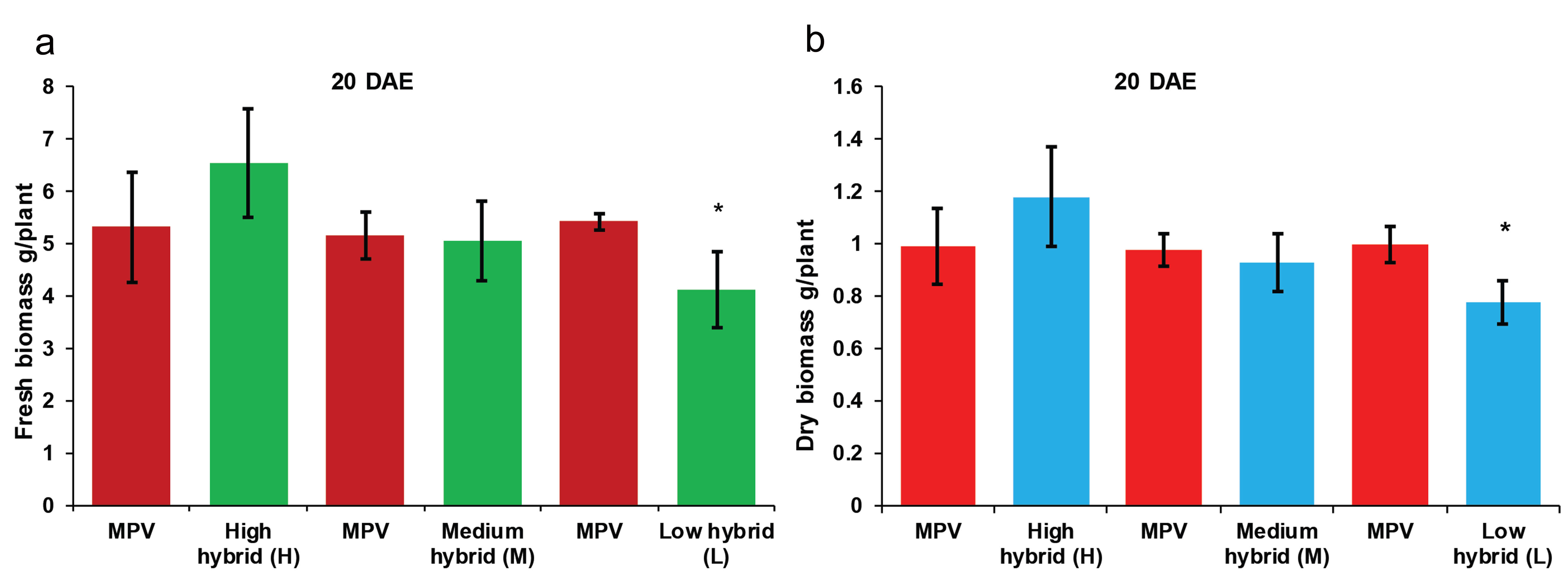

Supplement: Supplementary file 26 — Additional file 26: Figure S2. Phenotypic heterosis observed in all F1 hybrids at 20 days after emergence of seedling (DAE). Here * is used for significant difference at p < 0.0 a Fresh biomass in hybrids compared with their mid-parent value (MPV). b Dry biomass observed in hybrids compared with their MPV. [file 12864_2020_6561_MOESM26_ESM.png]

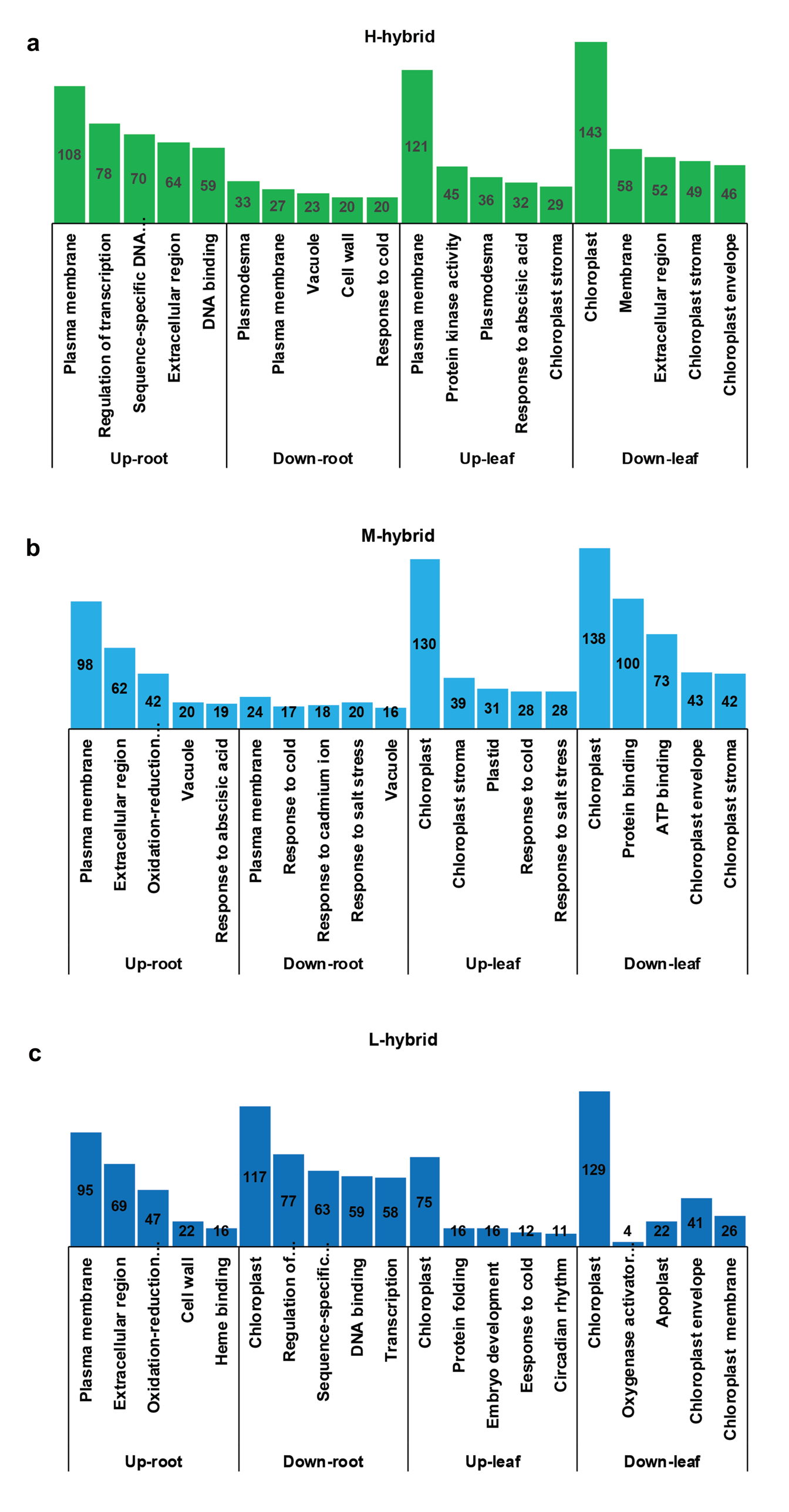

Supplement: Supplementary file 27 — Additional file 27: Figure S3. Most enriched GO terms of overdominant DEGs in root and leaf of F1 hybrids. a, b, and c shows GO terms with total number of genes for up and down overdominant DEGs of high (H), medium (M) and low (L) hybrids, respectively. Here, most enriched GO terms with p < 0.05 are only presented. [file 12864_2020_6561_MOESM27_ESM.png]

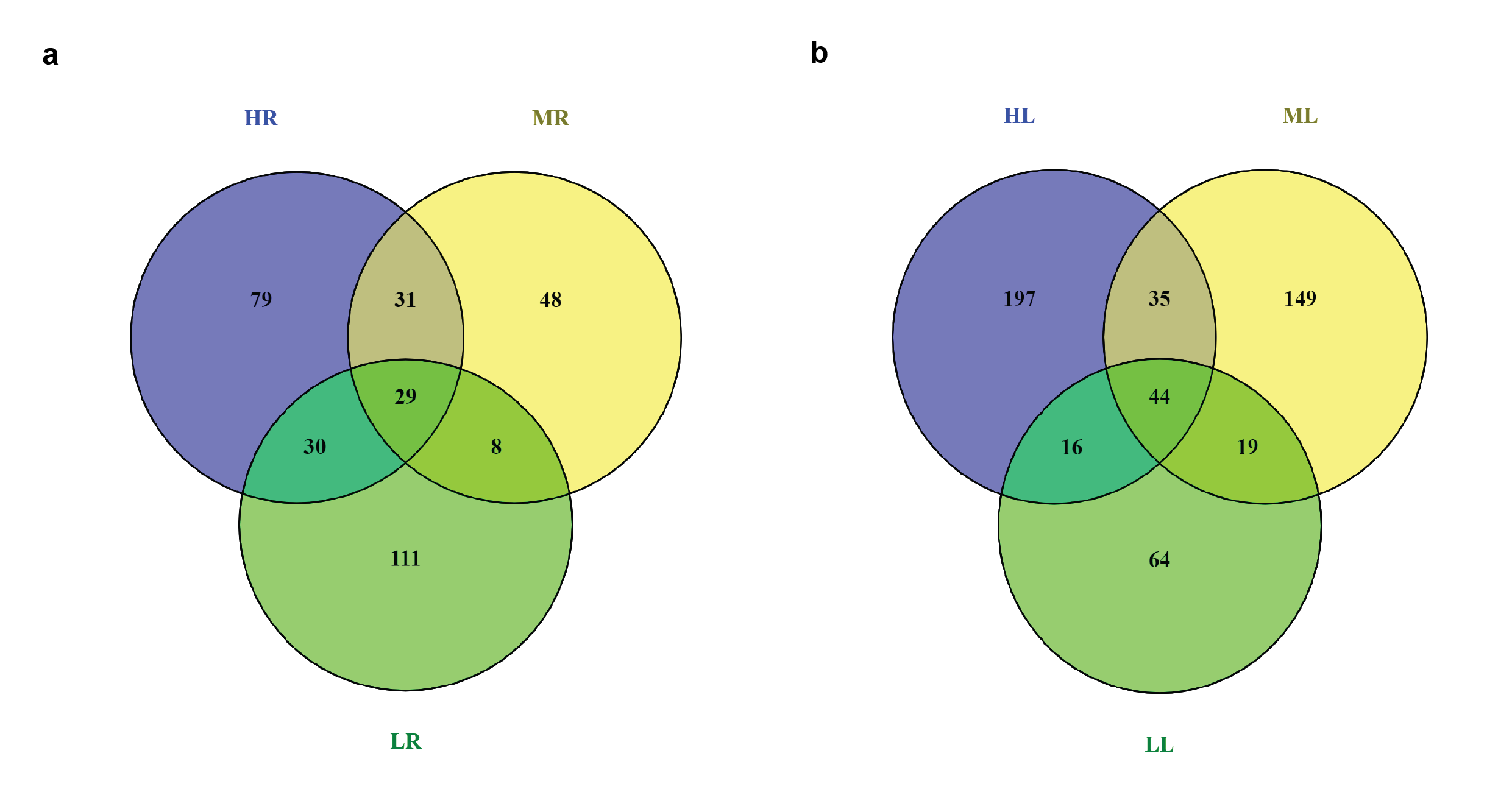

Supplement: Supplementary file 28 — Additional file 28: Figure S4. Venn diagram representing the comparison of overdominant genes between hybrids in root and leaf. L: Leaf, R: Root, H, M, and L represent high, medium, and low hybrids respectively. a Distribution of genes in root. b Distribution of genes in leaf. [file 12864_2020_6561_MOESM28_ESM.png]

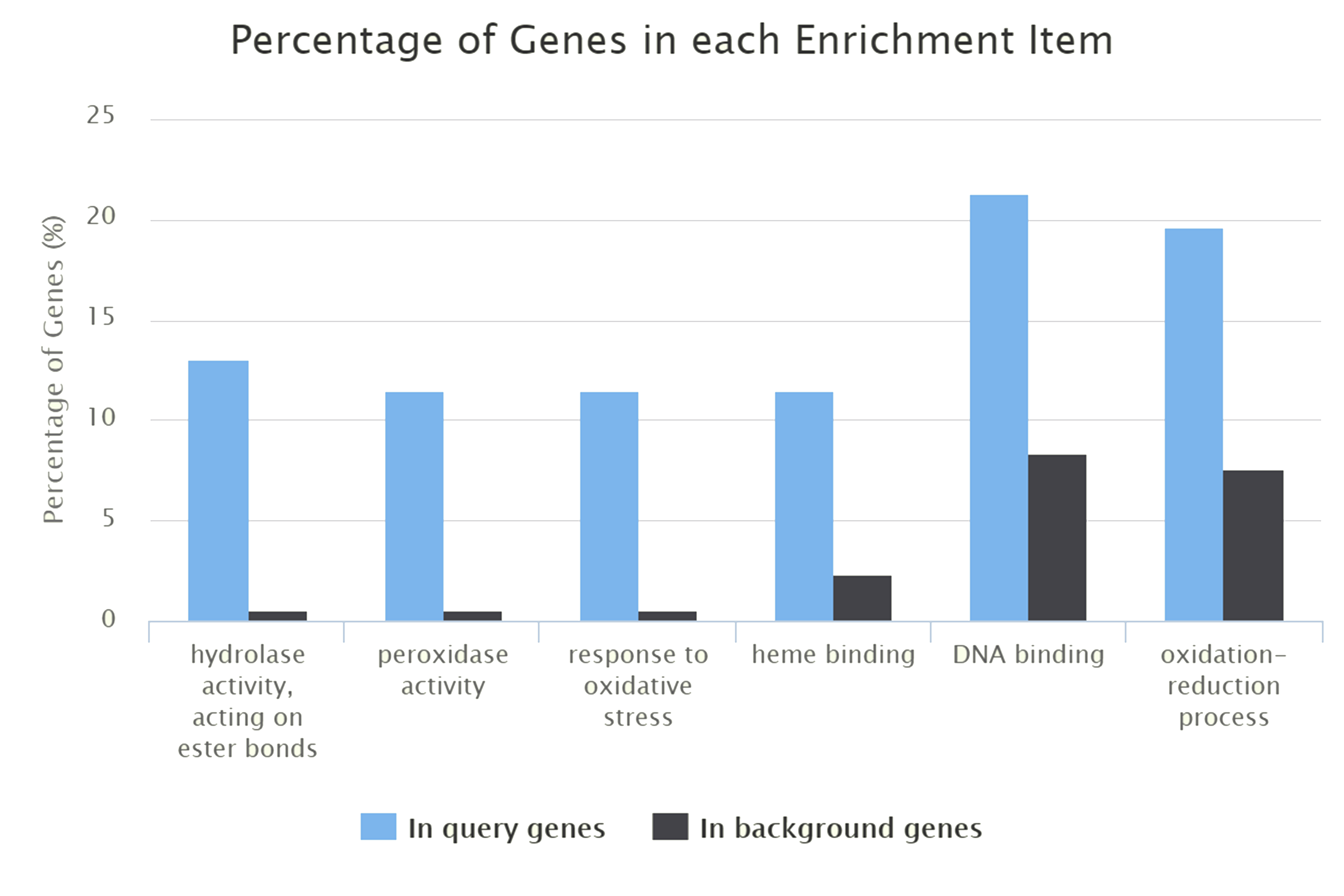

Supplement: Supplementary file 29 — Additional file 29: Figure S5. Enriched GO terms for genes that showed overdominant expressions in all hybrids relative to their parents in root and leaf. [file 12864_2020_6561_MOESM29_ESM.png]

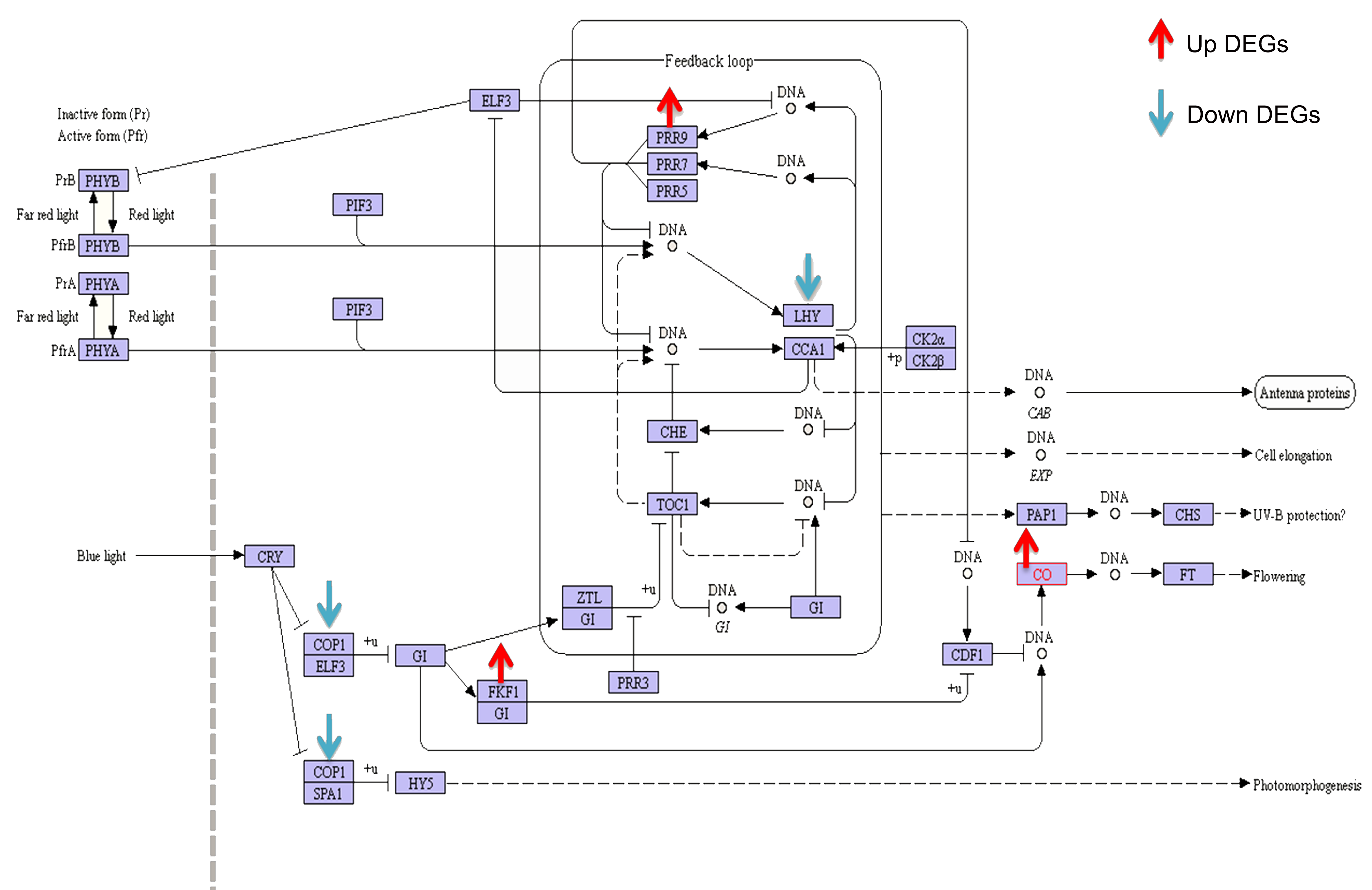

Supplement: Supplementary file 30 — Additional file 30: Figure S6. Functional annotations and mode of regulation for overdominant DEGs involved in circadian rhythm plant pathway. This figure is an interpretation of online available figure (https://www.kegg.jp/kegg-bin/show_pathway?ko04712+K12133). [file 12864_2020_6561_MOESM30_ESM.png]
